# Incidence and Prevalence of Polymyalgia Rheumatica (PMR): The Importance of the Epidemiological Context. *The Italian Case*

**DOI:** 10.3390/medsci7090092

**Published:** 2019-08-30

**Authors:** Ciro Manzo

**Affiliations:** Rheumatology Outpatient Clinic, Poliambulatorio “Mariano Lauro”, Sant’Agnello—Distretto Sanitario 59 (Penisola Sorrentina), ASL Napoli 3 sud, 80065 Sant’Agnello, Naples, Italy; cirmanzo@libero.it; Tel.: +39-081-533-1465; Fax: +39-081-533-1449

**Keywords:** polymyalgia rheumatica, incidence, prevalence, epidemiology, out-of-hospital public rheumatologist

## Abstract

Objectives: to evaluate incidence and prevalence rates of polymyalgia rheumatica (PMR) in Italy, depending on the epidemiological methodology used from time to time. Materials and Methods: A comprehensive literature search in MEDLINE and EMBASE was carried out. The following search terms were used: polymyalgia rheumatica, incidence, prevalence, epidemiology, general practitioner, family medicine, Italy. A search was also carried out in Google scholar using the search phrase: epidemiology of polymyalgia rheumatica in Italy. The period considered was between 1970 and March 2019. All articles containing data on incidence and prevalence of PMR in Italy were read in full. Reviews and non-original manuscripts were excluded as well as all the studies containing incidence and prevalence rates of giant cell arteritis (GCA), unless clearly distinct from data related to patients with PMR alone (isolated and pure PMR). Results: Five articles corresponded to inclusion and exclusion criteria. Two articles were excluded as they were review articles, and three articles were excluded because there were not clear data on incidence and prevalence rates of isolated PMR. Three articles reported data on the annual incidence of PMR (two of them published by the same group of investigators); two articles reported prevalence data. In one article, both incidence and prevalence were calculated. The annual rate of incidence of PMR was between 0.12 and 2.3 cases/1000 inhabitants aged over 50 years. In the two studies publishing prevalence data, they varied from 0.37% to 0.62%. The differences in incidence and prevalence rates were related to several factors such as the different set of diagnostic criteria used for identifying patients or the diagnostic difficulty for patients with atypical presentations, specifically those without raised erythrocyte sedimentation rate (ESR). In the study with higher annual rate of incidence and higher prevalence of PMR, the collaboration between general practitioner (GP) and the out-of-hospital public rheumatologist resulted in significantly different data than in the other studies. All the five articles presented data from monocentric cohorts. Conclusion: Very few Italian studies addressed the epidemiology of PMR. The contribution of a specific professional figure represented by the out-of-hospital public rheumatologist, present in the Italian National Health System and absent in other countries, can make the Italian experience unique in its kind.

## 1. Introduction

Polymyalgia rheumatica (PMR) is a common inflammatory disease affecting older adults. Bilateral shoulder and hip pain, often accompanied with neck aching, and morning stiffness lasting >45 min are typical manifestations of PMR. In many cases, the patient remembers the exact day and hour of the symptoms’ onset. Constitutional manifestations such as weight loss, fever of unknown origin, general discomfort and fatigue, and loss of appetite may complete the clinical picture [1,2,3,4]. The diagnosis of PMR still remains basically clinical, and no specific laboratory tests are available. Inflammatory markers (such as erythrocyte sedimentation rate (ESR) and C-reactive protein (CRP) concentrations) are usually raised at the time of diagnosis. However, normal ESR and CRP should not be a reason of exclusion for PMR [5,6,7].

There are several PMR-mimicking diseases and some of them can be recognized only through proper follow-ups [8,9]. In Table 1, the most relevant differential diagnoses are listed.

Giant cells arteritis (GCA) is closely linked to PMR: 40%–60% of GCA patients also have manifestations of PMR whereas 10%–16% of PMR patients can have manifestations of GCA. It is well-known that the association of PMR with GCA has significant therapeutic and prognostic consequences [10].

Among PMR-mimicking diseases, neoplasms deserve further investigation. The possibility that PMR may be a paraneoplastic syndrome is unanimously accepted [11,12], but the frequency with which this happens is still under discussion [13,14]. In fact, in 2010, Ji et al. examined the overall and specific cancer risks among Swedish subjects following hospitalization for PMR and GCA and noted that the risk of cancer was highest in the first year after hospitalization (of 3941 total cancer diagnoses, 783 (19.1%) were in the first year) [15]. In 2013, Muller et al., using data from the General Practice Research Database, highlighted that elderly patients with a PMR diagnosis were significantly more likely to receive a cancer diagnosis in the year after PMR diagnosis (313/667 cancer cases (69%)) [16]. In the experience of other investigators, this risk was significantly lower [17,18]. The type of diagnostic set (territorial, hospital, or university set, for example) and of adopted methodology (retrospective study, population database for example) represented the most differentiating elements. Moreover, the higher risk of cancer in the first year after PMR diagnosis observed by some investigators could express a misdiagnosis. Finally, the possibility that PMR associated with remitting symmetrical seronegative synovitis with pitting edema (RS3PE) may be a neoplastic warning has been also highlighted [19].

PMR onset peaks around the age of 75 and its prevalence increases until the age of 90, with a slight decrease thereafter: this trend by age groups is observed in all geographical areas, regardless of the latitude and socio-environmental characteristics of the population studied [20,21,22,23,24,25]. The possibility that PMR may onset in a centenarian man has recently been described [6]. The general practitioner (GP) is usually the first physician who examines the PMR patient, and many PMR patients are not referred to rheumatologists [26,27,28,29,30]. In a study from the UK only 44.4% of patients with PMR underwent specialist consultant evaluation [8]; in another study, this percentage dropped to 17% [26]. In Italy, the presence of a professional figure represented by the out-of-hospital public specialist (specifically, an out-of-hospital public rheumatologist) introduces an element of substantial differentiation with the recognition of a dividing line between GPs and centers of the so-called second or third level, totally absent in other nations [2,28,30].

## 2. Objectives

Primary objective: to evaluate incidence and prevalence of PMR in Italy.

Secondary objective: to evaluate the contribution of the out-of-hospital public rheumatologist in intercepting PMR patients.

## 3. Materials and Methods

A comprehensive literature search in MEDLINE and EMBASE was carried out on 14 March, 2019. The following main search terms were used: polymyalgia rheumatica, incidence, prevalence, epidemiology, general practitioner, family medicine, Italy. The search was then completed in Google scholar using the following search phrase: epidemiology of polymyalgia rheumatica in Italy. References from all selected studies were also examined. Exclusion criteria were: not Italian case studies, review and non-original articles, epidemiological data regarding GCA if they were not clearly distinguished from those related to pure PMR.

## 4. Results

We found only five studies that met these criteria (Table 2).

Two articles were excluded as they were review articles [23,25]. Three articles were excluded because there were no clear data on incidence and prevalence rates of isolated PMR [36,37,38].

In 1987, Salvarani et al. identified in the population of Reggio Emilia (few less than 170,000 inhabitants) 56 patient affected by PMR, and found a middle annual rate of incidence equal to 0,12/ 1000 inhabitants over 50 years of age [31]. In 1991, the same investigators published data related to the period 1980–1988. They identified 76 patients affected by PMR: 25 M, 51 F; relationship F/M = 2.04; 25% of them had an age among 70 and 79 years, at the time of diagnosis. In this second report, they confirmed the first annual rate of incidence [32]. The diagnosis of PMR was according to Healey’s criteria: (1) persistent pain from at least one interesting month at least two of the following areas: neck, scapular track, pelvic track; (2) morning stiffness lasting at least 1 h; (3) fast response to low doses (20 mg/d) of prednisone; (4) absence of other confounding diseases; (5) age >50 years; (6) ESR > 40 mm/first hour [39]. In both the first and the second report, the data referred to adult patients sent by their GPs.

In 2004, Salaffi et al. performed a study named MAPPING (MArche Pain Prevalence INvestigation Group) [33]. The first objective of this study was the evaluation of the prevalence of different musculoskeletal diseases in a primary care setting. The study was performed with the collaboration of 16 GPs and it was structured in a first phase in which a questionnaire was randomly sent to 3664 subjects of age >18 years; in a second phase, rheumatologists visited the patients which had answered to the questionnaire, and evaluated all the collected data. The diagnosis of PMR was according to Bird’s criteria, that is PMR was diagnosed if at least three were present among the following: (1) bilateral shoulder pain; (2) ESR > 40 mm; (3) clinical manifestations lasting at least two weeks; (4) functional impairment shoulders; (5) morning stiffness >1 h; (6) age at onset >65 years; (7) depression and/or loss of weight [40]. In the MAPPING study, the prevalence of PMR was of 0.37% in persons over 50 years of age.

In 2009, we published the data of prevalence and incidence of PMR in Massa Lubrense, a little town in the Surrentina peninsula with 13,500 inhabitants. In our study, a questionnaire was initially sent to all the 10 GPs working in Massa Lubrense. Of the citizens of Massa Lubrense, 95% were followed by these 10 GPS, at the time of our study. In this questionnaire, GPs reported how many PMR patients by year of diagnosis were present in their databases, with their demographic data, between 2000 and 2007. In a second step, data from the questionnaire were compared with those of “Mariano Lauro” outpatient-clinic database regarding PMR patients living in Massa Lubrense. The conflicting data were discussed with the GP, and all unconfirmed diagnoses were removed. In this study, the diagnosis of PMR was according to Healey’s criteria, but ESR < 40 mm at the time of diagnosis was not considered as an exclusion criterion if all other criteria were present. In this way, the study could also take in PMR cases with low ESR at the time of diagnosis. In the period 2000–2007, 89 new cases of PMR (36 M, 53 F; relationship F/M = 1.40) were diagnosed. We found an annual incidence of 2.3 cases/1000 persons aged over 50 years, and a prevalence of 0.62% [34].

More recently, in their CAMPO-RHE (Campobasso rheumatology) study performed in an inland area of central Italy having fewer than 100,000 inhabitants, De Socio et al. diagnosed in a two-year study period 12 new cases of PMR, and found an annual incidence of 0.27/1000 persons aged over 50 years [35]. Patients were classified using the 2012 American College of Rheumatology/ European League against rheumatism (ACR/EULAR) classification criteria [41]. As in the Salvarani et al. studies, also in this observational study the rheumatologists visited the patients sent to hospital outpatient clinic by their GPs.

The two studies performed by Salvarani et al. were the only ones from which it was possible to follow the incidence trend of PMR: in the period 1980–1988, no change was observed.

## 5. Discussion

According to our best knowledge, there are only five reports concerning the incidence and prevalence of PMR in Italy. The absence of an exemption code for this disease, the absence of a specific code for the population studies as it is for the N20 code in the United Kingdom, the absence of a project involving GPs and rheumatologists (as the PMR cohort study) [42], are all limiting factors.

As already highlighted, PMR is a disease mainly managed in primary care by GPs and PMR patients are sent to the rheumatologist mostly in the presence of diagnostic or therapeutic issues (e.g., atypical findings such as ESR < 30 mm/1 h, PMR scarcely responding to prednisone therapy, PMR with severe systemic manifestations). On the other hand, PMR is by no means a straightforward disease [8] and the level of the GP’s diagnostic accuracy is often low [30]. This could cause an underestimation in the incidence and prevalence of PMR [8,26,27].

In Italy, there is a specific professional figure represented by the out-of-hospital public rheumatologist, who is not present in other countries. He may represent a link between the GP and so-called “second” (hospital) or “third” (university) level. The capability of this specialist to intercept a large proportion of patients with PMR is directly proportional to the level of collaboration that has been established with the GPs. When this collaboration is present, more patients with PMR are also visited by the rheumatologist [28,30,34].

As highlighted in Table 2, we found a PMR prevalence approximately twice that in the MAPPING study. However, in the MAPPING study the prevalence was calculated as periodic prevalence, referring to the period April–June 2004, and in the Massa Lubrense study it was calculated as prevalence point at 30 September 2007. As highlighted by other investigators, the MAPPING study generated the lowest European estimate to date for PMR prevalence [43]. In our study, PMR prevalence was very similar to that observed up to 1991 and in 2015 in Olmest County, Minnesota [44]. As known, the population of this county is mainly composed of descendants coming from the Scandinavian peninsula. The Scandinavian ancestry may influence the distribution of PMR in some countries [45], and many Scandinavians live in Massa Lubrense for decades. Nevertheless, it was not possible to evaluate whether and how this presence could influence the data of our study.

The annual rate of PMR incidence in Massa Lubrense study was much higher than in other Italian studies. The design of the study may explain most of these differences. In particular: (1) an ESR < 30 mm was not considered an exclusion criterion, and (2) all PMR patients were evaluated (and not only those referred to our outpatient clinic).

As regards the first point, it has been more and more highlighted in the literature that in a sizable proportion of PMR patients—from 7% to 22%—ESR is not raised at the time of diagnosis [46,47]. These patients may represent a more benign subset of disease without constitutional manifestations [48,49,50]. As already highlighted, in the Massa Lubrense study, ESR < 40 mm at the time of diagnosis was not considered as an exclusion criterion if all other of Healey’s criteria were present. In this study, 12% of PMR patients had an ESR < 40 mm/1 h at the time of diagnosis. Up to today, the reasons why ESR can be normal/not raised in PMR are only speculative. In PMR a non-specific inflammatory reaction is triggered by innate immunity activation [51]. Innate immunity may trigger fever, general malaise, fatigue, and other constitutional manifestations. PMR patients with low ESR have a lower frequency of these compared to PMR with high ESR [48,50]. On the other hand, constitutional manifestations cause an increase in inflammatory indices [52]. In PMR patients, the absence of constitutional manifestations could be a result of interactions between innate and adaptive immunity within a specific genetic background [53]. What is certain is that a normal ESR in patients with otherwise clinical PMR leads the GPs astray, resulting in a serious delay in diagnosis or in misdiagnosis [5,7,8,27,28,30]. In all the five studies we considered, patients with atypical PMR associated with GCA were by definition excluded from the final evaluation.

As regards the second point, in the two studies performed by Salvarani et al., and in the CAMPO-RHE study, the rheumatologist visited the patients sent to hospital outpatient clinic by their GPs and no information was present regarding patients with PMR who had not been sent to any rheumatologist or who had been sent to other hospitals or outpatient clinics. In the MAPPING study, only patients who had responded to a questionnaire were examined by the rheumatologist. So, inclusion and referral bias could not be excluded in these studies. On the contrary, in the Massa Lubrense study, all patients with PMR were considered regardless of who (GPs or rheumatologist) had diagnosed PMR. As already highlighted, all the conflicting data between GPs databases and the Mariano Lauro rheumatological database were discussed with the GP, and all unconfirmed diagnoses were removed.

A final, interesting point for discussion is the assessment and the evaluation of incidence of PMR in Europe. We know that the incidence rates of PMR is very different from country to country and from geographical area to geographical area, with lower annual incidence in Southern European countries. For example, the annual incidence rate of PMR in different areas of Denmark, over the period 1982–1994, was 0.41/1000 for persons aged 50 years and older [53]. More recently, in an English population-based study, the overall incidence rate of PMR was 0.95/1000 [54]. The two studies performed by Salvarani et al. [31,32] generated a low estimate for PMR incidence, similar to that observed, for example, in northwestern Spain where the annual incidence rate over the period 1987–1996 was 0.135/1000 persons aged over 50 [55,56]. Again, it deserves to be highlighted that the incidence rates of PMR we found in Massa Lubrense study were much higher than in European/non-Italian epidemiological studies. Differences in methodologies may explain such different data. For example, all these studies used hospital and/or university databases, which could have generated statistical bias [22,27,30]. Moreover, as already highlighted, the out-of-hospital public rheumatologist is a totally absent professional figure in the public health organization of other European countries [28].

## 6. Conclusions

As the Italian case highlighted, a different epidemiological perspective seems mandatory.

PMR is a disease mainly managed in primary care, but the level of the GP’s diagnostic accuracy is often low. A better collaboration between GP and out-of-hospital public rheumatologist could give epidemiological information significantly different from that currently available. In fact, many PMR patients may not be present in hospital and university databases, and the data we presented suggests that the incidence of PMR could be much higher than commonly thought.

From this point of view, all future epidemiological studies should also consider territorial data, in addition to hospital or university data.

## Figures and Tables

**Table 1:** Diseases with which a differential diagnosis has to be made. Signs and symptoms useful for a correct diagnosis.

Disease	Signs and Symptoms Useful for a Correct Diagnosis
Rheumatoid arthritis	Involvement of some joints of the hands (metacarpophalangeal II and III, and proximal interphalangeal), positive results of rheumatoid factor and anti-cyclic citrullinated peptide antibodies (ACPA), radiographic and ultrasound findings (erosive arthritis, periarticular osteoporosis).
RS3PE	Symmetric multiple synovitis, seronegative in rheumatoid factor and ACPA, causing boxing-glove swelling with pitting edema of hands and feet. Ultrasound findings: tenosynovitis of extensor tendon sheath.
Late-onset spondyloarthropathies, including ankylosing spondylitis and psoriatic arthritis	Inflammatory pain in the lumbar region; radiographic findings of sacroiliitis; psoriasis.
Late-onset systemic lupus erythematosus, sclerodermia, Sjogren’s syndrome, vasculitis	Inflammatory pain in the lumbar region; radiographic findings of sacroiliitis; psoriasis.
Late-onset systemic lupus erythematosus, sclerodermia, Sjogren’s syndrome, vasculitis	Presence of antinuclear antibodies, presence of antineutrophil cytoplasmic antibodies.
Idiopathic inflammatory myopathies (dermatomyositis, polymyositis)	Skin rashes, increased creatine kinase in the blood.
Scapulohumeral periarthritis, adhesive capsulitis (“frozen shoulder”)	Restriction of shoulder movements, even in passive; ultrasound and magnetic resonance imaging allow one to diagnose the specific inflammation. Inflammatory markers not raised.
Calcium pyrophosphate deposition disease	Monoarthritis; radiographic and ultrasound findings.
Paraneoplastic syndromes	Failure to respond to glucocorticoid therapy or frequent relapses must be considered as elements of suspicion. Furthermore, the presence of untypical clinical manifestations and of laboratory findings (among these, macrocytic anemia or bicytopenia), and familiarity for neoplasms should also be considered as warning.
Fibromyalgia	Inflammatory indices in their normal range, presence of tender points, widespread chronic pain.

**Table 2:** Italian epidemiological studies of polymyalgia rheumatica (PMR).

Authors	Year	Urban Area	Population	Setting	Criteria	Incidence	Prevalence
Salvarani et al. [31]	1981–1985	Reggio Emilia	169,950	hospital	Healey	0.12 **	?
Salvarani et al. [32]	1980–1988	Reggio Emilia	169,950	hospital	Healey	0.12 **	?
Salaffi et al. [33]	2004	Marche	20,882	primary care	Bird	?	0.37%
Manzo et al. [34]	2000–2007	Mass Lubrense	13,500	primary care	Healey, mod. *	2.3 **	0.621%
De Socio et al. [35]	2014–2016	Campobasso	100,000	hospital	EULAR/ACR	0.27 **	?

* ESR < 40 mm at the time of diagnosis was not evaluated as a non-diagnostic element if all other Healey’s criteria were present; ** annual incidence. Data for 1000 inhabitants aged over 50 years old.
